# Degenerate PCR Primers to Reveal the Diversity of Giant Viruses in Coastal Waters

**DOI:** 10.3390/v10090496

**Published:** 2018-09-13

**Authors:** Yanze Li, Pascal Hingamp, Hiroyasu Watai, Hisashi Endo, Takashi Yoshida, Hiroyuki Ogata

**Affiliations:** 1 Bioinformatics Center, Institute for Chemical Research, Kyoto University, Uji 611-0011, Japan; yanzeli@kuicr.kyoto-u.ac.jp (Y.L.); endo@kuicr.kyoto-u.ac.jp (H.E.); 2 Aix Marseille Université, Université de Toulon, CNRS, IRD, MIO UM 110, 13288 Marseille, France; pascal.hingamp@mio.osupytheas.fr; 3 Graduate School of Agriculture, Kyoto University, Kitashirakawa-Oiwake, Sakyo-ku, Kyoto 606-8502, Japan; hrhsys@gmail.com (H.W.); yoshiten@kais.kyoto-u.ac.jp (T.Y.)

**Keywords:** Megaviridae, meta-barcode, B family DNA polymerase (PolB), metagenome, *Mimiviridae*

## Abstract

“Megaviridae” is a proposed family of giant viruses infecting unicellular eukaryotes. These viruses are ubiquitous in the sea and have impact on marine microbial community structure and dynamics through their lytic infection cycle. However, their diversity and biogeography have been poorly characterized due to the scarce detection of Megaviridae sequences in metagenomes, as well as the limitation of reference sequences used to design specific primers for this viral group. Here, we propose a set of 82 degenerated primers (referred to as MEGAPRIMER), targeting DNA polymerase genes (*polBs*) of Megaviridae. MEGAPRIMER was designed based on 921 Megaviridae *polBs* from sequenced genomes and metagenomes. By applying this primer set to environmental DNA meta-barcoding of a coastal seawater sample, we report 5595 non-singleton operational taxonomic units (OTUs) of Megaviridae at 97% nucleotide sequence identity. The majority of the OTUs were found to form diverse clades, which were phylogenetically distantly related to known viruses such as Mimivirus. The Megaviridae OTUs detected in this study outnumber the giant virus OTUs identified in previous individual studies by more than an order of magnitude. Hence, MEGAPRIMER represents a useful tool to study the diversity of Megaviridae at the population level in natural environments.

## 1. Introduction

Viruses represent a non-cellular biological component of aquatic microbial communities [1]. By outnumbering cellular organisms in the same ecosystem [2,3], viruses exert top-down control on microbial community dynamics and drive carbon and nutrient cycling [4,5,6,7]. High-throughput sequencing approaches targeting viral or whole microbial communities have effectively uncovered genomic features and biogeography of viruses that infect prokaryotes (bacteria and archaea) and provided deep insights into their ecological functions [8,9,10]. However, much less is known for viruses that infect marine eukaryotes [11].

Of eukaryotic viruses, the recently proposed “Megaviridae” family is emerging as an important viral group in marine ecosystems [12,13]. Members of this viral family are often called giant viruses [14,15,16,17,18,19] given their large genome (370 kb–1.51 Mb) and particle (150 nm–1.2 µm) sizes. The Megaviridae family has been proposed to comprise two subfamilies [20]: “Megamimivirinae”, composed of viruses infecting amoebas [21] and microzooplankton [22], and “Mesomimivirinae”, which currently groups viruses infecting unicellular algae [23,24]. Megaviridae belongs to the proposed order “Megavirales”, composed of nucleocytoplasmic large DNA viruses (NCLDVs) [25]. Hereafter, we use these proposed taxonomic names for clarity and simplicity, although we acknowledge that these classifications have not yet been authorized by the International Committee on Taxonomy of Viruses (ICTV).

Recent studies revealed that members of Megaviridae are ubiquitously distributed in the sea with the abundance ranging from 10^3^ to 10^5^ genomes per milliliter of seawater [12]. They are transcriptionally active everywhere in the sunlit ocean and their transcripts dominate viral RNAs detected in metatranscriptomes of pico- and nano-planktonic eukaryotes [13]. However, viruses of Megaviridae and their DNA are much less abundant compared with other pico-sized organisms in the oceanic environments [12,16]. Therefore, it is difficult to appropriately assess the diversity of this clade with non-targeted (i.e., “shotgun”) metagenomic sequencing, since their genomes are greatly outnumbered by cellular genomes from which they cannot be separated by typical filtration methods. For example, Megaviridae, prokaryotes, and picoeukaryotes are preferentially collected together in the same 0.2–3 µm size fraction. To better understand the biogeography and ecological roles of Megaviridae, other complementary methods that allow investigation of their diversity at a finer scale, at the population or species level, are required.

Targeted approaches (i.e., amplicon-based methods) have long been used for this purpose, to characterize the diversity of large DNA viruses in the sea. For instance, Chen and Suttle [26] designed a set of primers targeting B family DNA polymerase genes (*polB*) of *Phycodnaviridae* and revealed five operational taxonomic units (OTUs) by restriction fragment length polymorphism analysis based on 33 cloned polymerase chain reaction (PCR) products from environmental samples [27]. Larsen et al. [28] designed a pair of degenerate PCR primers targeting the major capsid protein genes (*mcp*) of Megaviridae and *Phycodnaviridae*. These primers were combined with a high-throughput sequencing technology and used to document phylogenetic composition, diversity, and successional dynamics of these viral clades in a natural environment [29]. The DNA repair *mutS8* gene and *polB* were also used to assess the phylogeny of Megaviridae [30] and *Phycodnaviridae* [31], respectively. Overall, approaches based on a pair of degenerate primers can reveal more than 100 OTUs in either Megaviridae or *Phycodnaviridae* in the marine environment, thus being effective for studying these viruses. However, due to the high nucleotide sequence variations in even the most conserved viral protein coding genes [30], we suspected that a single PCR primer pair, even if it is degenerated, might not be universal enough to capture the true diversity of a NCLDV family in the wild.

Another difficulty in the design of novel primers for Megaviridae is the limitation of reference sequences due to the small number of characterized viruses in this family. However, the recent growth of deep metagenomic sequencing datasets, such as *Tara* Oceans metagenes [32], enable us to collect a wide variety of environmental sequences closely related to known members of Megaviridae. In this study, we developed a set of 82 pairs of degenerate primers to cover the highly diverse *polB* genes of marine Megaviridae. In addition to *polB* genes from known Megaviridae genomes, we used a much larger set of environmental Megaviridae *polB* sequences from the *Tara* Oceans metagenomes [32] to design the primers. The *polB* gene was selected because it is present as a single copy gene in all known Megaviridae genomes, and also because its homology with other viral and cellular *polB* sequences offers the possibility to build phylogenetic trees that can be used to distinguish Megaviridae *polBs* from *polBs* of other clades. We used this new set of primers, hereafter called MEGAPRIMER, to examine the richness of a natural Megaviridae community in a Japanese coastal seawater sample. We demonstrate that MEGAPRIMER is an efficient tool to characterize the richness of Megaviridae in a seawater sample at the species level.

## 2. Materials and Methods

### 2.1. Primer Design

**Figure 1:**
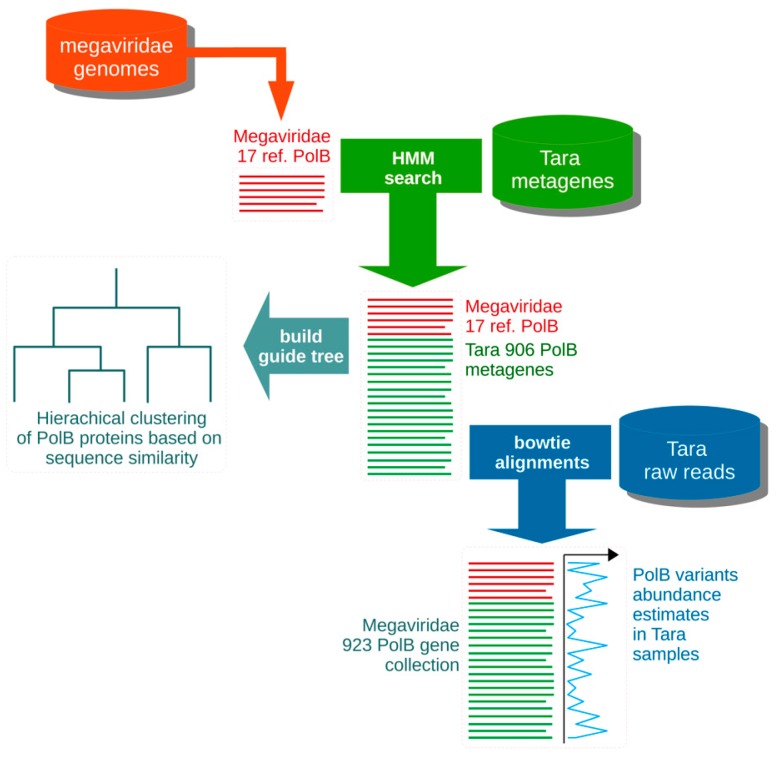
Schematic diagram of the building of the Megaviridae *polB* collection. This figure shows how the input data (reference Megaviridae genomes, *Tara* Oceans metagenes, and *Tara* Oceans raw reads) were used to produce a Megaviridae guide tree and a collection of 923 Megaviridae *polB* gene sequences.

**Figure 2:**
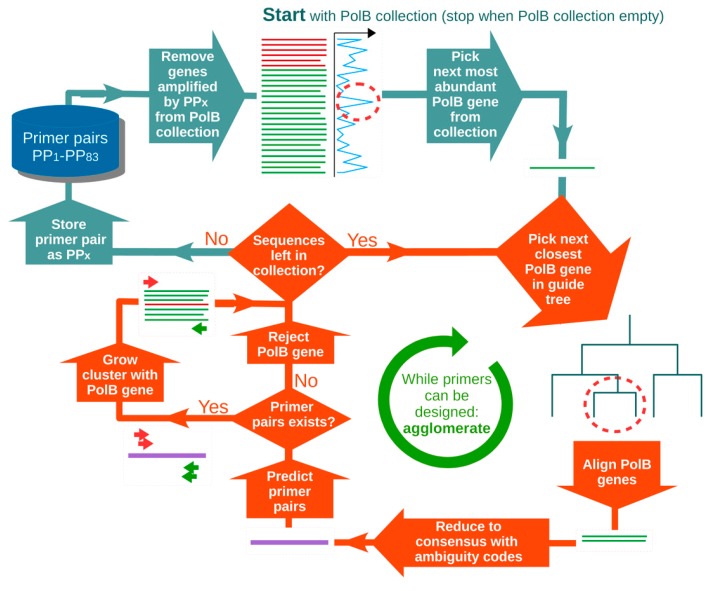
Schematic diagram of the design of the set of Megaviridae *polB* primer pairs. This figure shows the greedy agglomerative iterative procedure guided by the estimated environmental *polB* abundances (derived from read mappings) and the *polB* sequence similarities (encoded in the guide tree).

A general overview of the primer design strategy is provided in Figure 1 and Figure 2. A set of 17 reference PolB protein sequences translated from reference Megaviridae genomes (Table S1) were aligned with Multiple Alignment using Fast Fourier Transform (MAFFT) version 7.244beta (--thread -1 --genafpair --maxiterate 1000) [33]. Those 17 reference PolB sequences were obtained from all the 17 Megaviridae genomes that were available at the time of our primer design. The alignment of the corresponding 17 *polB* nucleotide sequences was obtained by threading the 17 reference gene sequences in the protein alignment using PAL2NAL version 14 [34]. Inspection of the nucleotide alignment suggested seven conserved domains (A–G, Figure S1), which might be suitable for designing oligonucleotide primers for PCR amplification. In order to enrich the 17-seed reference sequence alignment with additional diversity from the *Tara* Oceans metagenomes, we isolated six sub-regions (1–6) from the reference protein alignment and built six corresponding Hidden Markov Models (HMMs; Figure S1) using HMMER version 3.1b1 [35]. The primer design procedure described in Figure 2 produced the best results, meaning smaller number of primer pairs with best in silico PCR detection coverage of the *Tara* Oceans *polB* diversity. Therefore, when targeting the PolB domain G—which contains the location of AVS2 primer [26] and LSB-e primer [36], and does not show long insertions such as inteins in the reference sequences—the rest of the present procedure is described for the G domain only. The HMM N°6 identified 3537 putative *polB* genes out of a total 101,856,227 *Tara* Oceans metagenome assembled genes (European Nucleotide Archive (ENA) study PRJEB7988), of which 1230 were taxonomically assigned to the Megaviridae clade using Pplacer version 1.1.alpha16 [37] using a phylogenetic reference tree inferred from PolB reference sequences representing a large diversity of cells and viruses. The 1230 Megaviridae *polB* metagenes were clustered at 100% identity with CD-HIT version 4.6.1-2012-08-27-2 (-c 1.0 -G 1 -M 12000 -T 6 -d 0 -p 1) [38] yielding 990 non-redundant Megaviridae *polB* metagenes, including 232 full-length metagenes and 758 partial metagenes. The 990 PolB environmental proteins were aligned using MAFFT with the 17-seed reference Megaviridae PolB, and the sub-alignment region (346 amino acids) corresponding to domain G was extracted, yielding a Megaviridae PolB target collection of 923 sequences, supplemented with the PolB from *Ostreococcus tauri virus 1* (OTV1) to serve as an outgroup. Partial metagenes that did not span the full domain G were excluded. In order to guide the agglomerative primer design iterative strategy (Figure 2), a guide tree was built with MAFFT (mafft --retree 0 --treeout --localpair -reorder) using the domain G PolB 924 sequence alignment. An estimation of the abundance of each of the 923 Megaviridae *polB* metagenes in the ocean was calculated by counting the number of *Tara* Oceans reads (ENA study PRJEB7988) mapped to each sequence using Bowtie Version 2.2.4 (--no-hd --no-unal -f -S) [39] divided by the length of the *polB* sequence.

The agglomerative primer design procedure involved selecting the most abundant Megaviridae *polB* from the 923 sequences collection (Figure 2), called the seed, and building a cluster around it by iteratively aligning the next closest sequences from the collection using MAFFT (--maxiterate 1000 --globalpair). At each iteration, the cluster protein alignment served as a template to thread the corresponding nucleotide alignment using PAL2NAL. The clustered nucleotide alignment was then reduced to a single consensus nucleotide sequence, when appropriate with standard ambiguity codes, suitable for submission to optimized primer predictions using PRIMER3 version 3–2.3.6 [40], targeting the central region of domain G at position 105 to 214 in alignment. To further complement the PRIMER3 internal parameters, such as optimal primer melting temperatures of 52 °C, 20 mers optimal length, and maximal 8 degenerated positions, we added an additional constraint by filtering out PRIMER3 proposed primers with a degeneracy above 256. The sequence cluster was thus grown by agglomeration—in the order of sequence similarity—of Megaviridae *polB* sequences that led to successful primer pair predictions, and rejection of the others from the cluster. After all the *polB* collection had been tested for the current cluster, the successful primer pair was stored as PP1–PP83, and the sequences of the cluster were removed from the initial *polB* collection, as well as any other sequences of the collection that matched both primers using dreg from EMBOSS Release 6.6.0 [41]. This process was repeated with a new cluster seeded with the next most abundant *polB* sequence in the collection until the collection was empty.

The procedure yielded 83 primer pairs that in silico amplified 100% of the initial 923 Megaviridae *polB* sequences, which represents 97.4% of the abundance weighted 990 *Tara* Oceans Megaviridae *polB* metagenes. PP62 was excluded from the primer pair set as it only recognized two of the 923 *polB* metagenes that branched with the OTV1 outgroup and likely did not belong to the Megaviridae group. The set of 82 primer pairs was used to check for specificity by in silico PCR amplification of RefSeq Release 70 (Mar 03, 2015) using EMBOSS primersearch (-mismatchpercent 0 -auto -warning). Only nine virtual amplicons were detected, all against Megaviridae genomes (NC_021312 *Phaeocystis globosa virus* strain 16T, NC_014637 *Cafeteria roenbergensis virus* BV-PW1, NC_023639 *Mimivirus terra2*, NC_023640 *Megavirus terra1*, NC_020232 *Megavirus lba* isolate LBA111, NC_014649 *Acanthamoeba polyphaga mimivirus*, NC_016072 *Megavirus chiliensis*, and NC_020104 *Acanthamoeba polyphaga moumouvirus*). We named the resulting set of 82 degenerate primer pairs MEGAPRIMER version 1.

### 2.2. Sampling and DNA Extraction

A seawater sample (4 L) was collected from a 5 m depth at the entrance of Osaka Bay, Japan (N 34°19′28″, E 135°7′15″) on 30 October 2015. Seawater was prefiltered through 142 mm 3.0 μm-pore polycarbonate membrane (Merck, Billerica, MA, USA) and then 1 L of the filtrate was filtered sequentially through a 0.22 μm-pore Sterivex filtration unit (SVGV010RS, Merck, Billerica, MA, USA). After filtration, the 0.22 μm filtration unit was transferred to −80 °C. DNA was extracted from the stored filtration units by using a xanthogenate-sodium dodecyl sulfate (SDS) method according to a previously published protocol [42].

### 2.3. PCR Amplification and Sequencing

Each primer pair was used in a separate PCR amplification. Overhang adapters were appended at the 5′ end of each primer (forward overhang: 5′TCGTCGGCAGCGTCAGATGTGTATAAGAGACAG, reverse overhang: 5′GTCTCGTGGGCTCGGAGATGTGTATAAGAGACAG) according to the Illumina 16S Metagenomic Sequencing Library Preparation guide [43]. PCR was performed for cycles of 30 s at 94 °C, 30 s at 52 °C, and 30 s at 72 °C with a final elongation step of 4 min at 72 °C using KAPA HiFi HotStart Readymix (KAPA BIOSYSTEMS, Wilmington, MA, USA). PCR was run for 32 cycles for all amplifications except for two primer pairs, PP16 and PP45, for which we additionally performed PCR with 26 and 38 cycles to investigate the effect of the number of PCR cycles on sequencing results. The resulting PCR products were visualized using gel electrophoresis. To distinguish reads from different PCR products, multiplex barcodes were attached to the amplicons. DNA concentration of library was determined by Qubit BR dsDNA Assay Kit (Thermo Fisher Scientific, Waltham, MA, USA). The molarity was calculated according to Illumina’s 16S library preparation guide, by presuming the size of amplicons to be 500 bp as the amplicon size of reference sequences ranged from 374 bp to 590 bp including adapters and primers (about 112 bp). All amplicons were diluted to 2 nM and mixed. Further dilution yielded 8 pM final libraries. Two runs of sequencing were performed using a MiSeq platform with MiSeq V3 (2 × 300 bp) reagent kits (Illumina, San Diego, CA, USA) and with a spike-in of PhiX at 20% to serve as an internal control.

### 2.4. Quality Control and Merging

Prior to merging the paired-end reads, the reads underwent quality-control screening. We discarded reads if the average quality was below 5 or if the length was shorter than 40 bp. If one of the paired-end reads was removed in this step, the mate was also discarded. Primer sequences in the paired-end reads were identified using Cutadapt version 1.11 [44] with an error-tolerance rate of 10%. Only those paired-end reads harboring both forward and reverse primers were retained, and primer sequences were removed from the reads. Paired-end reads were then merged using FLASh [45]. FLASh, with an error tolerance rate of 10% and a minimum overlap of 100 bp, was applied three times in a sequential manner. First, FLASh was used with the “innie” orientation option to merge paired-end reads. Second, paired-end reads that were unmerged by the first round of FLASh were processed with “outie” orientation option. Partial primer sequences that could not be removed by the initial Cutadapt processing were identified at this stage and removed. Third, FLASh was applied again with the “innie” option for the remaining unmerged paired-end reads, but this time with an additional low-quality tail processing prior to merging. We trimmed low-quality tails of reads with the use of the “SLIDINGWINDOW” method in Trimmomatic v.0.33 [46]. We tested various window sizes (1 to 60 bp) and window qualities (Q1 to Q40) for Trimmomatic, and automatically chose the best parameters that produced the largest number of merged reads. Unmerged reads were discarded. Chimeric sequences were identified using USEARCH [47] implemented in qiime 1.9.1 [48], and were removed.

### 2.5. Taxonomic Assignment

Merged reads were deduplicated using CD-HIT-EST v4.6.1 [38] with a clustering threshold of 100% identity. The longest sequence in each cluster served as the representative sequence for the following analysis. Nucleotide sequences of the representative sequences were translated into amino acid sequences along all three possible reading frames on the direct strand, from forward to reverse primer, using Biopython v.1.68 [49]. Translated sequences from reading frames containing stop codons were discarded. Amino acid sequences were searched against a database containing PolB sequences from various organisms and viruses, including Megaviridae PolB reference sequences used in the design of MEGAPRIMER, with the use of BLASTP v.2.5.0 [50]. Sequences best hitting to Megaviridae PolBs (either known or metagenomic sequences) with an E-value lower than 1 × 10^–5^ were retained for further analysis. To further verify the taxonomic assignment to Megaviridae, the amino acid sequences were analyzed using Pplacer [37]. The amplicon sequences that were not assigned to the Megaviridae branches were discarded at this stage. Since the positions of forward and reverse primers along the Megaviridae *polB* reference sequences were slightly different among primer pairs, the extremities of the amplicon sequences were further trimmed to retain only regions that were common across all amplicons. For this purpose, the amplicon amino acid sequences assigned to Megaviridae were multiply aligned against the 923 reference Mimiviridae PolB sequences using MAFFT v.7.309 [33] with -add --6merpair, and were trimmed to retain only the common regions. The corresponding regions of nucleotide sequences were also prepared. The bioinformatics pipeline developed in the present study for sequence preprocessing and taxonomic assignment of MEGAPRIMER meta-barcodes was named Megaviridae Amplicon Processing System (MAPS).

### 2.6. Clustering and Phylogeny

To assess sequence richness, we clustered the trimmed amplicon sequences taxonomically assigned to Megaviridae using CD-HIT-EST with varying nucleotide sequence identity thresholds from 90% to 99%. Rarefaction curves were generated using the matplotlib package [51] by excluding singleton OTUs. For phylogenetic tree reconstruction, we selected the most abundant 1000 OTUs generated at 90% amino acid sequence identity cutoff, and reconstructed a phylogenetic tree based on the representative sequences of the OTUs (i.e., the longest sequence in each OTU) with an additional 20 PolB sequences from known Megaviridae, and 9 distantly related sequences from *Phycodnaviridae* used as an outgroup. The phylogenetic tree was generated using FastTree default setting (JTT + CAT model) and visualized using the Python ETE3 package [52].

### 2.7. Data Availability

The MAPS pipeline with associated data as well as sequences, phylogenetic trees, and gene alignment data used for primer design are provided in the following FTP site: ftp://ftp.genome.jp/pub/tools/MEGAPRIMER.

### 2.8. Accession Number

The sequence data generated in this study are accessible from DDBJ under the accession number DRA006230.

## 3. Results

### 3.1. In silico Assessment of MEGAPRIMER

We designed 82 pairs of degenerate primers (MEGAPRIMER, PP1–PP83) based on an extended set of 921 Megaviridae *polB* sequences (Table S2, Figure S1). Seven of these primer pairs were found to match the 17 *polBs* from sequenced Megaviridae genomes (PP25, 28, 47, 57, 61, 70, and 83; Table S3). In silico PCR, with 10% of maximum mismatches allowed, indicated that some of the primer pairs can amplify *polB* genes from Megaviridae that were recently sequenced and were therefore not included in the design of MEGAPRIMER (Table S3). These Megaviridae are *Aureococcus anophagefferens virus*, *Bodo saltans virus* [53], and *Klosneuvirus* (KNV) [54]. In contrast, none of the primer pairs corresponded to the *polB* sequence from Catovirus (CTV), which is a close relative of KNV and was also not included in the MEGAPRIMER design. In silico PCR against all the 921 reference *polB* sequences and their phylogeny indicated that reference sequences that can be amplified by a specific primer pair are not necessarily similar to each other along their length (Figure S2).

To assess the power of non-targeted shotgun metagenomic approaches in capturing the richness of Megaviridae, we used a previously published deeply sequenced marine metagenomic dataset composed of 243 sets of sequence assemblies [32]. When we counted the occurrence of the amplicon regions targeted by MEGAPRIMER in these samples, 25 genotypes per sample on average (from 0 to 289 genotypes per sample) were revealed (Table S4).

### 3.2. MEGAPRIMER Effectively Amplified Megaviridae polB

DNA extracted from a seawater sample served as the template for the PCR amplification. PCR products with sizes comparable to the expected sizes were observed by gel electrophoresis for 58 out of the 82 tested primer pairs (including PP16 and PP45) at 32 PCR cycles (Figure S3). For PP16 and PP45, additional positive PCR reactions were also obtained by applying 26 and 38 PCR cycles. We thus obtained a total of 62 positive PCR products. Sequencing of the PCR products produced 16,677,495 raw paired-end reads, of which 9,377,470 were successfully merged (Table 1, Table S5). Out of the merged reads, 8,440,486 (90.0%) were predicted to be of Megaviridae origin based on our Pplacer phylogenetic placement pipeline (100–526 bp; 309.9 bp on average). These corresponded to 60 PCR products. Note, two PCR products, PP64 and PP77, did not yield validated Megaviridae *polB* sequences. The remaining 936,984 (10.0%) merged reads were discarded at this stage of analysis; a large majority (99.5%) of these sequences did not show similarity to *polB* sequences from cellular organisms or viruses.

**Figure 3:**
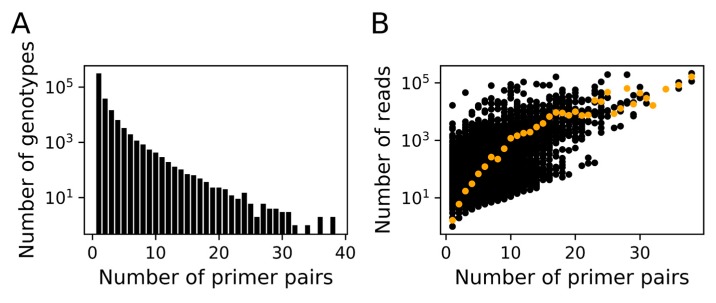
Genotypes represented by Megaviridae *polB* meta-barcodes. (**A**) Number of identified genotypes are plotted against the number of primer pairs that amplified individual genotypes. (**B**) Number of reads that support individual genotypes are plotted against the number of primer pairs that amplified corresponding genotypes. Average number of reads for each group of genotypes are shown by an orange dot.

After trimming the extremities, the length of the reads ranged from 9 to 273 bp (210.6 bp on average; 99.99% were >150 bp). The merged reads were then grouped into 381,722 genotypes by clustering at 100% sequence identity. Reads supporting a genotype were found to result from one or more PCR reactions (i.e., a single genotype could be amplified by different primer pairs), although most genotypes (313,133; 82%) were revealed by a single PCR reaction. The number of identified genotypes decreased with increasing number of primer pairs that amplified individual genotypes (Figure 3A), whereas the number of reads supporting individual genotypes increased (Figure 3B).

### 3.3. Richness of Megaviridae OTUs

**Figure 4:**
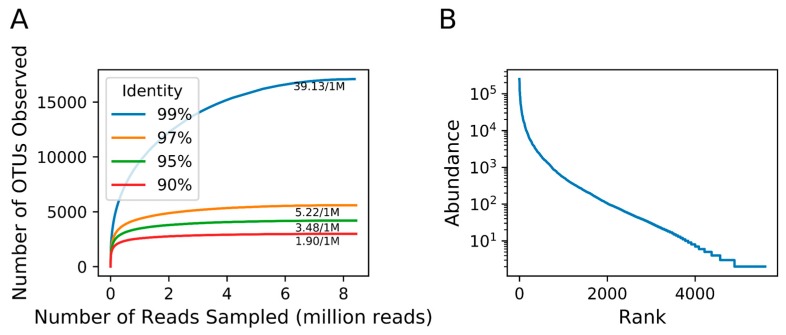
Megaviridae operational taxonomic units (out) richness. (**A**) Rarefaction curves for Megaviridae *polB* OTUs with various DNA sequence identity cutoffs. Proximity to saturation is indicated by weak slopes at the end of each rarefaction curve. For instance, an increase in 39.13 OTUs per resampling of one million reads is noted as 39.13/1M. (**B**) Rank abundance curve of Megaviridae *polB* OTUs at 97% DNA sequence identity. *Y*-axis represents the number of reads supporting individual OTUs.

The 8,400,486 Megaviridae *polB* sequences were grouped into OTUs using different nucleotide sequence identity cutoffs. After excluding singletons, we obtained 17,086, 5595, 4195, and 2984 OTUs for 99%, 97%, 95%, and 90% identity cutoffs, respectively (Table 2). Rarefaction curves indicated that the number of OTUs reached close to the plateau under the sequencing depth realized in this study for each clustering cutoff (Figure 4A and Figure S4). The most abundant OTU at 97% identity in our dataset contained 249,997 sequences (2.96%), whereas the average number of sequences in each OTU was 1507 (Figure 4B).

For PP16 and PP45, three PCR cycles were tested. The total number of sequences assigned to Megaviridae increased with increasing PCR cycles (Table S5; PP16: 24,706, 132,792 and 293,415 sequences with 26, 32 and 38 cycles, respectively; PP45: 24,399, 256,248, and 307,426 sequences with 26, 32, and 38 cycles, respectively). Although the number of sequences differed substantially between the tested cycles, the 100 most abundant OTUs were 99% shared between PCR conditions for PP16, and 98% shared for PP45 (Figure S5).

### 3.4. Phylogeny of Megaviridae polB Meta-Barcodes

**Figure 5:**
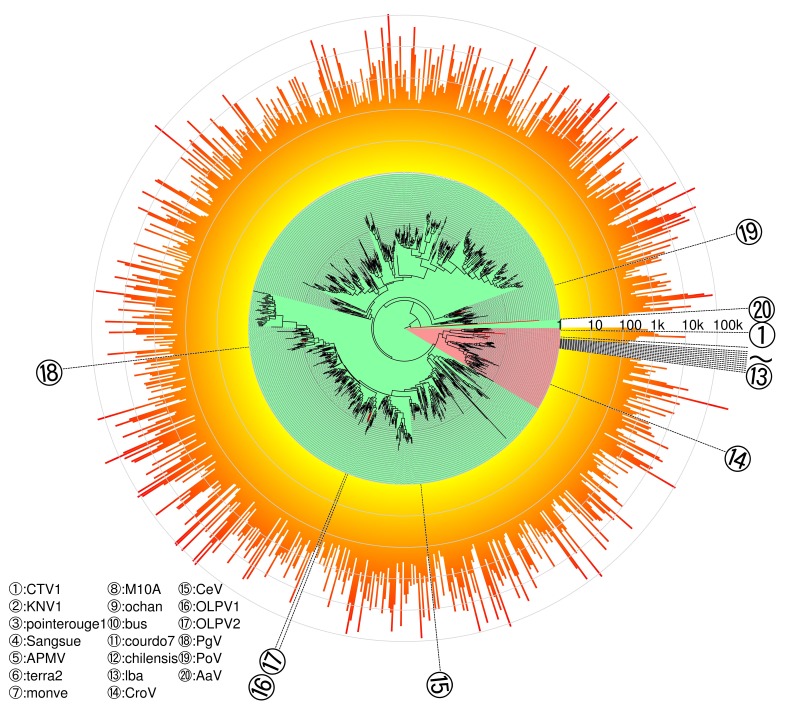
Phylogenetic tree of PolB meta-barcodes. Maximum-likelihood phylogenetic of Megaviridae PolB meta-barcodes with additional known Megaviridae sequences. Branch lengths are scaled. The tree is rooted by nine *Phycodnaviridae* sequences, which are not shown in this figure. Leaves are either meta-barcodes (black) or reference Megaviridae PolBs (red). Background colors of the tree indicate either putative Megamimivirinae (pink) or putative Mesomimivirinae (green). Lengths of orange bars outside the tree represent OTU abundances (number of reads, log scaled). CTV1: *Catovirus* (KY684084); KNV1: *Klosneuvirus* (KY684109); AaV: *Aureococcus anophagefferens virus* BtV-01 (NC_024697); BsV: *Bodo saltans virus* NG1 (MF782455); OLPV1: Organic Lake phycodnavirus 1 (HQ704802); OLPV2: Organic Lake phycodnavirus 2 (HQ704803); CeV: *Chrysochromulina ericina virus* isolate 01 (EU006632); PoV: *Pyramimonas orientalis virus* isolate 01 (EU006633); hirudovirus_Sangsue: *Hirudovirus* strain Sangsue (KF493731); megavirus_bus: *Mimivirus* Bus strain RTM2 (JF979178); megavirus_courdo7: *Megavirus* courdo7 isolate Mv13-c7 (JN885991); moumouvirus_ochan: *Moumouvirus ochan* (JQ063130); moumouvirus_monve: *Moumouvirus monve* isolate Mv13-mv (JN885998); MIMI_terra2: *Terra virus* 2 TAO-TJA (GU265562); MIMI_pointerouge1: *Mimivirus pointerouge1* strain Pr1 (JF979167); megavirus_lba: *Megavirus lba* isolate LBA111 (NC_020232); APMV: *Acanthamoeba polyphaga mimivirus* (NC_014649); megavirus_chiliensis: *Megavirus chiliensis* (NC_016072); moumouvirus_M10A: *Acanthamoeba polyphaga moumouvirus* (NC_020104); PgV: *Phaeocystis globosa virus* strain 16T (NC_021312); and CroV: *Cafeteria roenbergensis virus* BV-PW1 (NC_014637).

**Figure 6:**
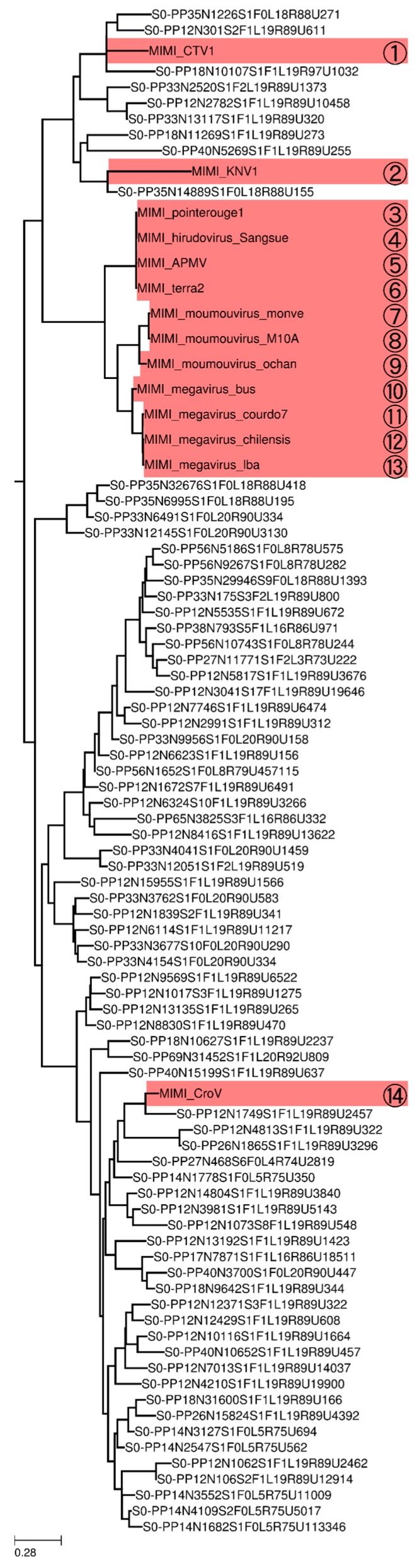
Phylogenetic sub-tree of PolB meta-barcodes. A magnified view of the phylogenetic tree around the Megamimivirinae group in Figure 5 is presented. The branch lengths are scaled. Reference genomic genes are indicated by the corresponding numbers in Figure 5.

Our phylogenetic analysis of representative sequences (OTUs at 90% amino acid sequence identity) revealed that MEGAPRIMER successfully amplified sequences close to known Megamimivirinae, shown by the branches in the pink area of Figure 5, such as PolBs of *Cafeteria roenbergensis virus* and recently sequenced Klosneuviruses (numbers 1, 2, and 14 in Figure 5, see Figure 6 for detail). Regarding Mimiviruses, only PP2 produced visible bands among four primer pairs (PP2, PP47, PP74, and PP83) that could theoretically amplify *polBs* of this virus group (Table S3, Figure 5). This may be due to a low concentration of this virus group in the tested sample, and consistently, no OTUs were detected inside the clade of Mimiviruses (numbers 3 to 13 in Figure 5 and Figure 6). As for the group of Mesomimivirinae, including viruses infecting unicellular algae (i.e., branches in the green area of Figure 5), dozens of diverse clades were revealed by the phylogenetic tree. The number of reads for OTUs dramatically varied inside the clades (over 100-fold), but showed rather similar profiles between clades; namely, no clades showed an exceptionally large or small number of reads relative to other clades. There was no strong correlation between the clades and primer pairs that amplified the sequences in the clades (Figure S6). In other words, sequences in a clade were generated by many primer pairs, and single primer pairs amplified sequences of different clades.

## 4. Discussion

To assess Megaviridae community structures in environmental samples, we developed MEGAPRIMER, a set of 82 pairs of degenerate primers targeting a conserved PolB C-terminal region. We used a large set of marine metagenomic data for the design of the primers to cover the wide genetic variations expected in Megaviridae *polB* genes. By applying the primers to a microbial DNA sample from coastal seawater, we detected 5595 OTUs at 97% DNA sequence identity for Megaviridae *polB*. The fact that many of the identified genotypes were revealed by a single pair of degenerate primers (Figure 3A) suggests that the combination of multiple pairs of degenerate primers was effective at broadening the detection of the diversity of Megaviridae. A previous study proposed that diverse marine unicellular eukaryotes potentially represent hosts of Megaviridae [16,55]. The high complexity of the Megaviridae community in a single sample parallels the complexity of eukaryote community in the marine environment (typically > 10,000 OTUs [56]), thus being consistent with the previous proposal.

Of the 82 degenerate primer pairs, 58 produced amplicons that were visualized by gel electrophoresis for the tested marine microbial DNA sample. Most of these primer pairs were found to be specific to Megaviridae *polB* genes (90.0% specificity, Table 1). Rarefaction curves for the meta-barcodes from the amplicons individually reached a plateau, suggesting that a single run of MiSeq was large enough to reveal the diversity of Megaviridae in the sample. The remaining 24 pairs of primers did not show clear bands in the agarose gel. Possible explanations are that the target *polB* genes of those primer pairs were not present in the tested sample or that amplicons were not sufficiently abundant to be visualized by gel electrophoresis. Additionally, we also tested different PCR cycles (26, 32, and 38) for PP16 and PP45. We found that there was no notable difference in the major Megaviridae OTU compositions across different PCR cycles, underlining the reproducibility of our method. It should be noted that meta-barcoding by MEGAPRIMER may not be quantitative because individual *polBs* can be amplified by various numbers of primer pairs (Figure 3 and Figure S2), though it would still allow presence/absence comparisons of OTUs across multiple samples for ecological analyses.

Our approach, which leverages sequence diversity present in marine metagenomes, achieved more than one order of magnitude wider Megaviridae OTU coverage compared to previously reported methods. In previous studies, degenerate primers were designed to target *polB*, *mcp*, and *mutS8* genes of *Phycodnaviridae* and/or Megaviridae and used to characterize their existence and diversity in environmental samples [26,28,30]. In an early study, Chen et al. [27] applied a *polB*-specific primer pair to seawater samples from the Gulf of Mexico. Larsen et al. [28] also designed a degenerate primer pair for *mcp* genes of *Phycodnaviridae* and Megaviridae and applied the pair to Norwegian fjord waters. Both studies elucidated the divergent nature of *Phycodnaviridae* and Megaviridae in the oceanic environment. However, only a few OTUs were detected in these studies due to the limitation of sequencing methods that were available at that time. Recent adaptations of such targeted methods to high-throughput technologies have revealed greater diversities in Megaviridae and other NCLDVs [29,30,57]. For example, Johannessen et al. [29] used the 454 platform to sequence *mcp* gene amplicons derived from Norwegian fjord water samples. They acquired several thousand reads per sample, which were clustered into 51–186 OTUs at 95% amino acid sequence identity. Recently, another set of degenerate primers targeting *mcp* genes of algal Megaviridae (i.e., Mesomimivirinae) was designed and used against samples collected from a brown tide [58]. Sanger sequencing of clones of PCR products produced 475 *mcp* gene fragments, which were classified into 145 OTUs at 97% nucleotide sequence identity.

In our experiment, each primer pair generated on average 338 non-singleton OTUs among the successful PCR products (Table S5). Some of our rarefaction curves saturated only after subsampling of several hundred thousands of reads (Figure S4), suggesting the necessity of the deep sequencing, as was performed in our study, to accurately evaluate the diversity of Megaviridae. When all the amplicon reads were pooled together, we revealed a high richness of Megaviridae (over 17,086 OTUs at 99% and 5595 OTUs at 97% identity). These values are much greater than those estimated from previous targeted approaches or those based on the existing non-targeted but deeply sequenced marine metagenome data. As we do not have shotgun metagenomes from the tested Osaka Bay sample, we used *Tara* Oceans metagenomic data; we could detect only 25 genotypes per sample on average (and 289 genotypes per sample at most) for Megaviridae from the marine metagenomes (Table S4). Although the number of OTUs depends on various aspects of samples and methods, such as the nature of sampled environment, sequencing depth, sequencing region, and pore-size of the membrane filter, our study is the first to identify thousands of Megaviridae OTUs at 97% nucleotide sequence identity clustering cutoff from a single sample. Therefore, our meta-barcoding approach—MEGAPRIMER combined with MiSeq sequencing—represents a method to characterize the genetic diversity of environmental Megaviridae at an unprecedentedly high resolution.

MEGAPRIMER detected not only sequences that were closely related to the few known Megaviridae members, but also a vast space of distantly related unidentified members (Figure 5). Based on our phylogenetic analysis, 99.5% of the meta-barcodes had no known close relative at a cutoff of 80% amino acid sequence identity. A total of 91% amplicon sequences appear to be classified in the algae-infecting group of Megaviridae (i.e., Mesomimivirinae). The remaining 9% of sequences were assigned to the Megamimivirinae group represented by the amoeba-infecting mimiviruses. This bias toward Mesomimivirinae is consistent with a previous observation by Hingamp et al. [12]. The virions of some Mesomimivirinae, such as *Chrysochromulina ericina virus* and *Phaeocystis globosa virus*, have been reported to have smaller diameters than the 0.22 µm pore size used in this study [14,59]. Therefore, our filtration procedure might not have captured all the virions of this group of viruses present in the water sample. It should be also noted that the design of MEGAPRIMER was largely based on published marine metagenomic sequences, where Megamimivirinae is known to be scarce [12]. Therefore, the present version of MEGAPRIMER may not be optimal for the coverage of the potential diversity of Megamimivirinae in the environments not represented by the currently available marine metagenomes. Nevertheless, our method detected sequences related to Klosneuviruses of the Megamimivirinae (Figure 6). Klosneuviruses were only very recently identified [53,54], and their sequences were not included in the design MEGAPRIMER. This result justifies our approach to use metagenomic data to design primers for the detection of previously uncultured viruses.

Our multiple primer approach may have a drawback to consume a larger quantity of template DNA for PCR reactions compared to the use of a single pair of primers when amounts of available DNA are limited. Furthermore, our primers were tested only against one sample. There could be large variations in the diversity and abundance of Megaviridae depending on season and geographical location [12,29]. Therefore, the MEGAPRIMER approach proposed in this study should be further evaluated with different environmental samples. Our preliminary study indicates that MEGAPRIMER can be used to identify multiple Megaviridae OTUs from other aquatic samples (manuscript in preparation). Our future work also includes the assessment of mixtures of some of the 82 primer pairs into primer cocktails for reducing the amount of DNA needed for meta-barcoding. Notwithstanding some technical improvements to be possibly achieved, we think that MEGAPRIMER already represents a useful tool to characterize the diversity, biogeography, and ecological roles of Megaviridae, a widespread and abundant group of giant viruses in the marine environment. Finally, the strategy we employed in designing MEGAPRIMER, namely the use of environmental sequences to design not a single but multiple pairs of degenerate primers, may also be applied to other viral or cellular genes with high genetic variation.

## Figures and Tables

**Table 1:** Overview of number of analyzed sequences.

Raw	High Quality	With Primers	Merged	BLASTP Validated	Pplacer Validated
16,677,495	11,217,759	10,211,698	9,377,470	8,475,525	8,440,486

**Table 2:** Number of operational taxonomic units (OTUs) at each clustering threshold.

OTUs	Clustering Threshold			
	99%	97%	95%	90%
Total number of OTUs	75,923	13,243	6123	3426
Number of singleton OTUs	58,837	7648	1928	442
Number of non-singleton OTUs	17,086	5595	4195	2984
Slope at the tail of rarefaction curve (OTU/1,000,000 reads)	39.13	5.22	3.48	1.90
Number of sequences included in non-singleton OTUs	8,381,648	8,432,837	8,438,557	8,440,043

## Supplementary Materials

The following are available online at http://www.mdpi.com/1999-4915/10/9/496/s1, Figure S1: Alignment of 17 “Megaviridae” *polB* reference nucleotide sequences with consensus agreement, Figure S2: In silico detection of genomic and metagenomic ‘Megaviridae’ *polBs* by MEGAPRIMER, Figure S3: Gel electrophoresis of PCR products, Figure S4: Rarefaction curves for individual primer pairs, Figure S5: Number of OTUs shared by different PCR cycles for PP16 and PP45, Figure S6: Relationships between read acquisition for OTUs and primer pairs, Table S1: Seventeen reference genome sequences used in primer design, Table S2: Sequences of MEGAPRIMER, Table S3: In silico detection of ‘Megaviridae’ *polB* by MEGAPRIMER, Table S4: Number of genotypes of “Megaviridae” from *Tara* Oceans metagenomic data, Table S5: Number of sequences and OTUs for individual primer pairs.
