# Supplementary material for: Degenerate PCR Primers to Reveal the Diversity of Giant Viruses in Coastal Waters
Source: Viruses. 2018 Sep 13;10(9):496. doi: 10.3390/v10090496 (PMC6163766; doi:10.3390/v10090496)
Supplement: Supplementary file 1 [file viruses-10-00496-s001.pdf]

Supplementary Materials

Degenerate PCR primers to reveal the diversity of giant viruses in coastal waters

Yanze Li[1,•], Pascal Hingamp[2,•], Hiroyasu Watai[3], Hisashi Endo[1], Takashi Yoshida[3], Hiroyuki Ogata[1,*]

[1] Bioinformatics Center, Institute for Chemical Research, Kyoto University, Uji 611-0011, Japan
[2] Aix Marseille Université, Université de Toulon, CNRS, IRD, MIO UM 110, 13288, Marseille, France
[3] Graduate School of Agriculture, Kyoto University, Kitashirakawa-Oiwake, Sakyo-ku, Kyoto 606-8502, Japan
[•] These authors equally contributed to this study.
[*] Correspondence to Hiroyuki Ogata, ogata@kuicr.kyoto-u.ac.jp.

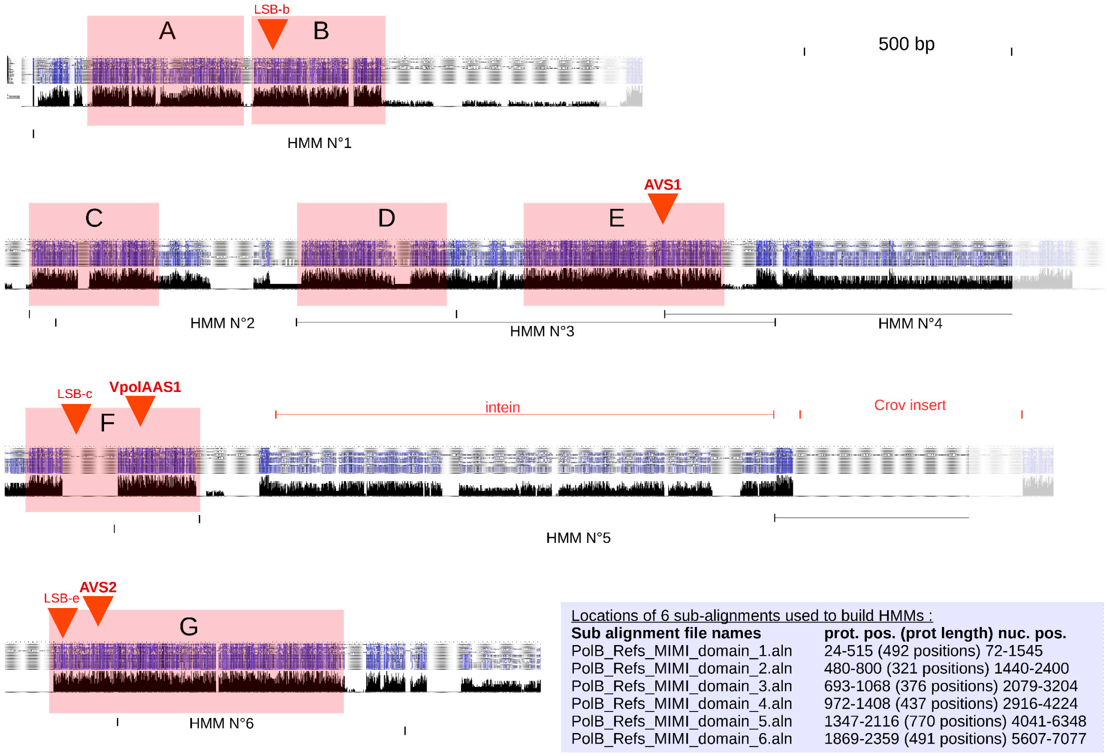

Figure S1. Alignment of 17 'Megaviridae' *polB* reference nucleotide sequences (top blue tracks) with consensus agreement (bottom black histograms). The location of seven conserved domains A-G are indicated by pink boxes, while the position of the six sub-alignments used to build HMM1-6 are indicated by black segments. The position of *polB* primers previously used in the literature are indicated by red triangles (Chen et al. 1995, La Scola et al. 2010, Clerissi et al. 2014), whilst the position of the *polB* intein and large CroV INDEL are indicated by red segments.

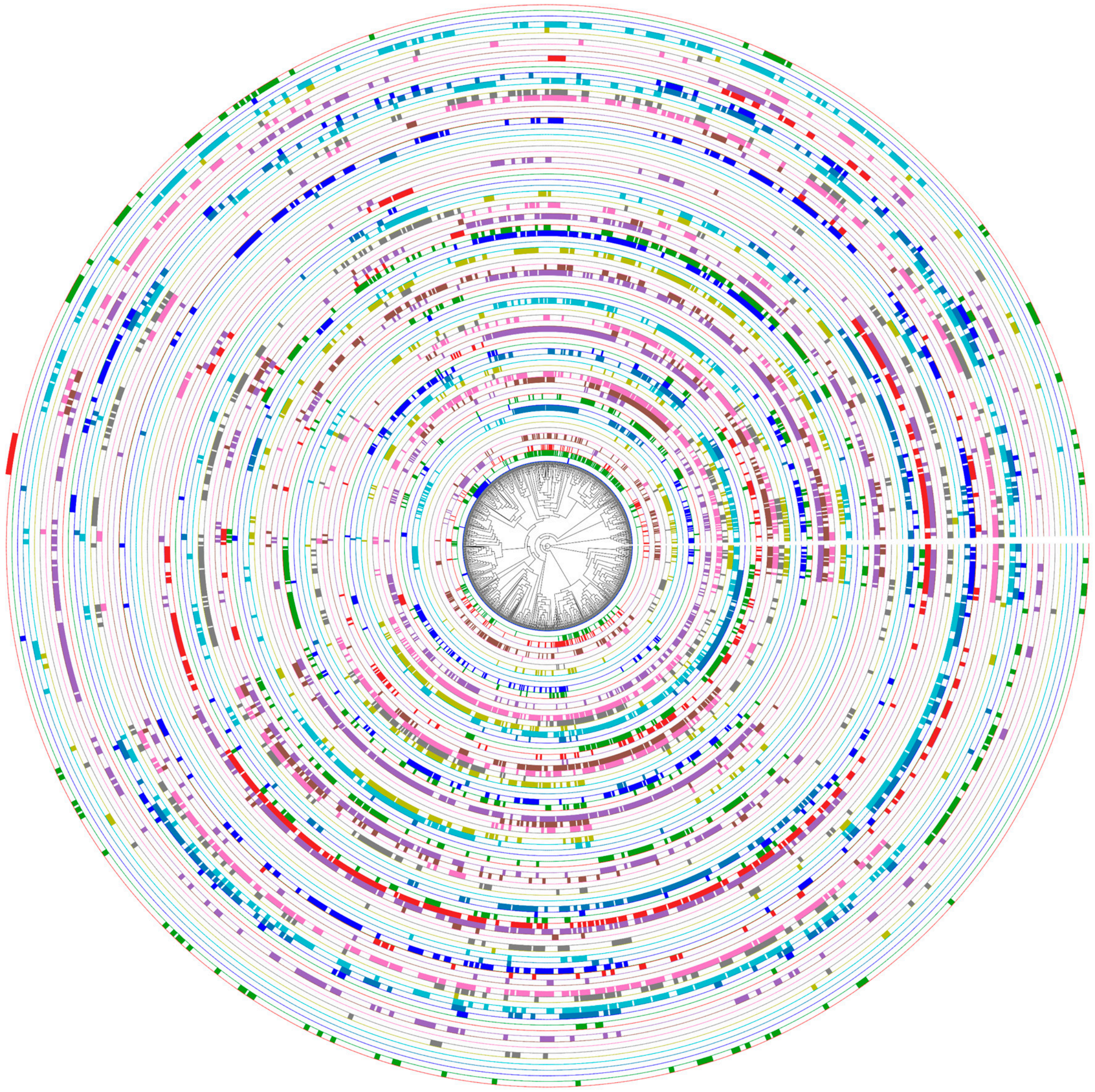

Figure S2. *In silico* detection of genomic and metagenomic ‘Megaviridae’ *polBs* by MEGAPRIMER. *In silico* PCR experiments were performed against 21 ‘Megaviridae’ *polBs* (17 references, AaV, BsV, KNV1 and CTV1) and 923 ‘Megaviridae’ *polB* metagenes (921 ‘Megaviridae’-like and 2 OtV-like) from *Tara* Oceans using 82 primer pairs with 10% of maximum mismatches allowed. Rings outside the phylogenetic tree represent primer pairs (PP1 to PP83 from inside to outside). Colored part of the rings indicates positive *in silico* PCR results.

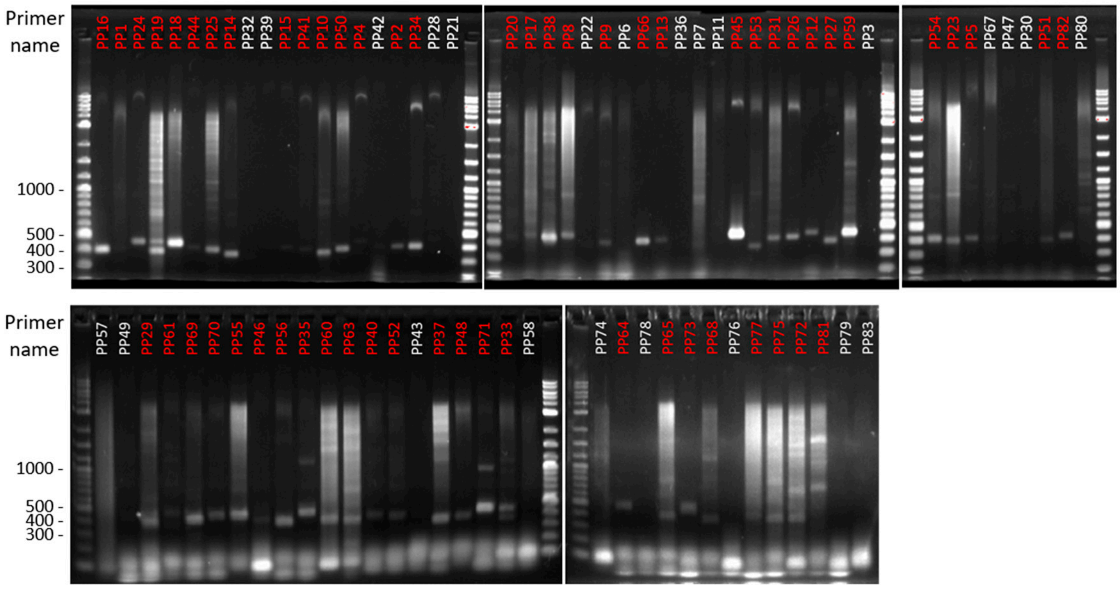

Figure S3. Gel electrophoresis of PCR products. PCR products with visible bands (red labels) were sequenced, while others (white labels) were discarded.

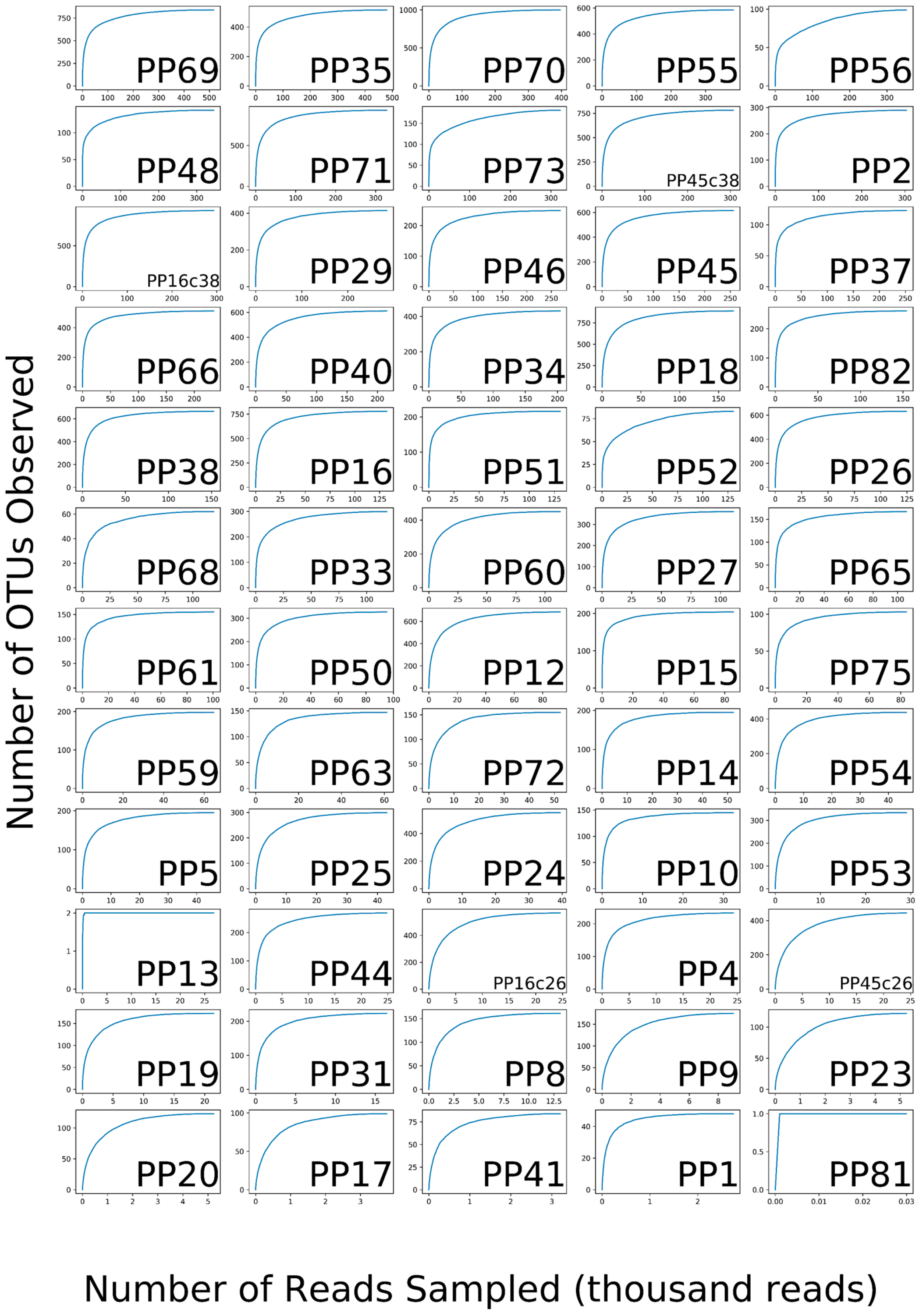

Figure S4. Rarefaction curves for individual primer pairs. This figure shows rarefaction curves for 60 samples for which high quality 'Megaviridae' *polB* sequences were generated. Sequences are clustered at 97% DNA identity.

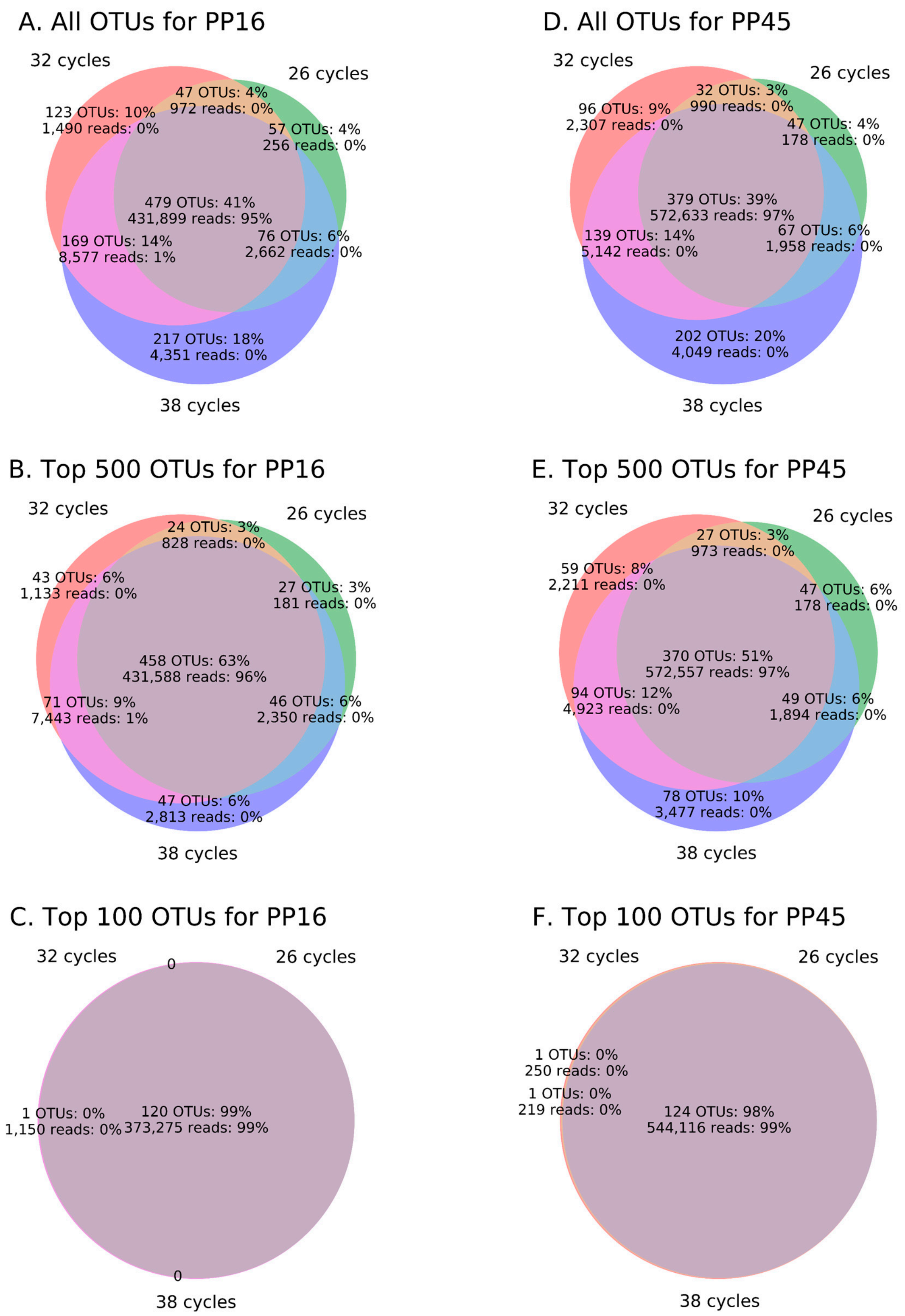

Figure S5. Number of OTUs shared by different PCR cycles for PP16 (A, B and C) and PP45 (D, E and F). Venn diagrams correspond to those for all OTUs (A, D), top 500 abundant OTUs (B, E) and top 100 abundant OTUs (C, F). OTUs were generated by grouping at 97% DNA sequence identity.

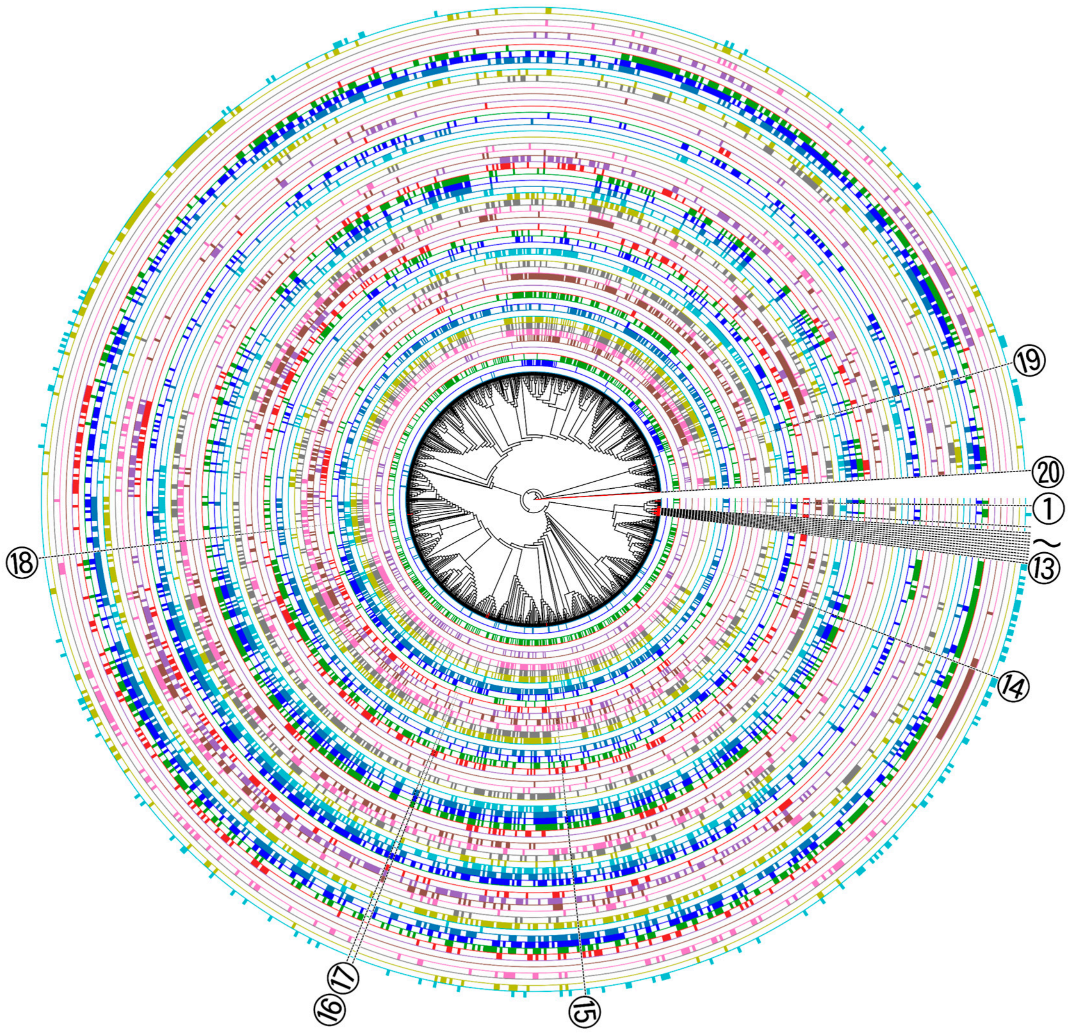

Figure S6. Relationships between read acquisition for OTUs and primer pairs. Same phylogenetic tree as in Figure 5 was used in the central part of the figure. Rings outside the phylogenetic tree represent primer pairs (PP1 to PP83 from inside to outside). Colored part of the rings indicates acquisition of reads for the corresponding primer pairs and OTUs (90% amino acid sequence identity). Reference genomic genes are indicated by the corresponding numbers in Figure 5.

Table S1. Seventeen reference genome sequences used in primer design

**Virus name**	**Access number**
Catovirus	KY684084
Klosneuvirus	KY684109
Aureococcus anophagefferens virus isolate BtV-01	NC_024697
Bodo saltans virus strain NG1	MF782455
Organic Lake phycodnavirus 1	HQ704802
Organic Lake phycodnavirus 2	HQ704803
Chrysochromulina ericina virus isolate 01	EU006632
Pyramimonas orientalis virus isolate 01	EU006633
Hirudovirus strain Sangsue	KF493731
Mimivirus Bus strain RTM2	JF979178
Megavirus courdo7 isolate Mv13-c7	JN885991
Moumouvirus ochan	JQ063130
Moumouvirus monve isolate Mv13-mv	JN885998
Terra virus 2 TAO-TJA	GU265562
Mimivirus pointerouge1 strain Pr1	JF979167
Megavirus lba isolate LBA111	NC_020232
Acanthamoeba polyphaga mimivirus	NC_014649
Megavirus chiliensis	NC_016072
Acanthamoeba polyphaga moumouvirus	NC_020104
Phaeocystis globosa virus strain 16T	NC_021312
Cafeteria roenbergensis virus BV-PW1	NC_014637

Table S2. Sequences of MEGAPRIMER.

Name	Forward primer	Reverse primer
PP1	DCGHAARAARGGHTATRTTGG	WGATTGWGGTTTRTTWCCNGGR
PP2	WGAATATGARAAAACHTTYTATCC	MATTCTMTCNGCHARWACTTTATG
PP3	CCACAYGATYTDGARTATGARAARAC	WARNAYAWWATGWGCAAT
PP4	YCCWCATAARYTWGAATAYGARAARAC	YGATTGDGGYTTATTKCCWGGRY
PP5	CCACAYGATSYWGARTAYGARAARACATT	KATCWGCHARHACCTTGTGD
PP6	RAARCGDTATGTBGGRATGY	YTTYGGRTTCTTRTABCCACTNC
PP7	RTATGARAARACNYTNATGCC	RTGWCCDATTTGCAWNGGR
PP8	NGCNCAAGAAGCNGGNGAA	DGCDARWACTTTATGWGCDAT
PP9	YAARAARMGATATGTDGGHAAT	TTGHGGNGCAYTWCCWGGRTCTC
PP10	RAARAGRTAYACDGGDGATAAAT	WGGRTTYCTYTCNGCCATWCKATC
PP11	VACCACATKAYYTDGAATW	WATAWAWACAWANGGWAYTCT
PP12	WCCACAYGAYYTAGARTAYGARAARAC	YTTRGGTTTATTWCCWGSRTCTCTH
PP13	YAYAAACCDCAAGCWYTWAATTAYGA	WGCYAAWACTTTATGTGCWA
PP14	NAARAARCGWTATGTDGGW	WCCWATTCTATCDGCTARWAC
PP15	GATAYACNGGNGAYAAATATGAR	DGCATANGGDATHCKATCATT
PP16	RGAATATGARAARACATTY	WGCYAABACTYTATGDGCDAT
PP17	RGAATAYGARAARACWTTTWKGCC	HATTCTATCACCWGVDCYWGGTT
PP18	DCAYGAYTTRGARTATGARAARAC	HGATGGTTTATTNCCNGGRTC
PP19	RTAYGARAARACVTTYATGCC	HACTTTRTGNGCDATTTGH
PP20	RTATGARAARACNYTNATGCC	GGTTTNGGYTTRTTHCCHGGGTC
PP21	RGMTAYRTTGGWAAYAAATAYGAAW	KAYRTAWACAWANGGWATTC
PP22	RTATGARAARACRYTNATGCC	DATYTGATTNGGATTYTTRTAATMAC
PP23	HAARAAAMGRTAYGTWGGWATG	VGGYTTATTACCDGRRTCGCGY
PP24	NCARGAYYTDGAATATGAAAARAC	TCATTKGGTTTNGGTTTRTYWCCHGG
PP25	YGATYTWGARTAYGARAARACATTY	YAAWACYTTRTGYGCDATTTGY
PP26	NCAAGAYTTRGARTATGARAARAC	HCCNATTCTATCNGCYAAYAC
PP27	NAARAARMGATATGTNGGWATG	GGWTCTCTTTTHSCHATYCTATCHGC
PP28	TATGARAAAACATTYATGCC	DATAWABACAWAWGGHA
PP29	RAARMGATATRYVGGAGATAARTATGA	HCCHGGRTCTCKTTCHGCCATKC
PP30	AAAAACGWTATACKGGMMTRTTRTATGAAK	WGCRAGAACTYTATGTGSRATW
PP31	YTGAARAARCCWCAYGATYTRGAR	YARYACTTTATGNGCDATY
PP32	RATATGTHGGWAATYTDTATGAR	HARNACTTTATGWGCWATY
PP33	YCCWCAWRATTTAGARTATGAAAAA	KCATTRCCDGGRYYTCTTKCTTT
PP34	WCCWCAYGAYTTRGARTAYGAAAARAC	GCHARNACCTTRTGHGCAAT
PP35	YTGGARTATGARAARACATTY	WAYACRWAYGGAATTCTATC
PP36	WCATGAYYTRGARTATGARAARAC	RAADGGDATHCKATCTCC
PP37	DCCATTTATCYTKTTYTCAAARAAR	DACYTTATGWGCWATCTGRTCHGGA
PP38	DGARTATGARAARACRTTYTGGCC	WGGYTTATTWCCWGGDTCTCTTTY
PP39	WAARAARAGRTATGTNGGN	DGTTTGWATATAHAYRAAHGG
PP40	TTTCCWCAYGATYTDGAATATGARAARAC	RATYCTWTCTGCYARDACWAC
PP41	DGGTGADAARYATGAAH	DGGWATWCKATCATTTGVTTTHGGTT
PP42	WTTAGARTATGARAARACRTTYTRKCC	MATTCTRTCYGCYAAWACYTTRTGW
PP43	MGDTATKTTGGWAATAAATATGAA	TCAATAWAWMMATAWGGWAYTCTRTCA
PP44	RCAYGATYTDGARTATGARAARAC	VATYCTATCTGCHARNAC
PP45	SCACCDCAYGATTTRGAATATGARAA	WGGTTTATTWCCHGGGTC

PP46	DCCWCAYGATYTRGARTATGARAAAACA	HACYTTATGWGCWATACTYTCRGGA
PP47	WAARAARARRTAYGTKGGAAAY	WGGKKYAKWWCCAGGRTCTC
PP48	YATTTTAYCCWTTYATTCTWATWTCT	KATCRGCTAAYACYTTRTGKGCWATM
PP49	WAARAARMGATAYGTAGGWRAY	RAAWGGDATYCTRTCATTWAY
PP50	NCAYGATYTDGAATATGARAARAC	HACTTTRTGNGCDATTTGM
PP51	MGATATGTKGGWAWKYTATATGAA	KCTSGGTKYATTKCCWGRATC
PP52	YGTTGGAATGTTRTATGAASAM	WAYAWAARCAWATGSWATKC
PP53	WAARAARMGRTATGTWGGHATG	GCHARHACTTTRTGHGCAATM
PP54	KCCRCAYGAYYTGGARTAYGARAA	BGCCAAWACYTTRTGHGCDAT
PP55	WCCRCAYGAYTTRGARTATGAAAARAC	WAGGCTTRTTKCCVGGATC
PP56	ATCCRTTTTGYATTTTAAGTAARAARMGW	TCATTCTRTCWGCTAAACAWACRTGWGC
PP57	NGCAMARGAAGCNGGYSA	RCAWAWGGWATTCKATCNCC
PP58	RTATGTRGGDATGTTRTATGAG	YAWAYGCAATMCGRTCYCC
PP59	RGAATATGARAAAACNYTDATGCC	HCCHGGYTTYGGYTTATTTCCWGG
PP60	BAARAAGMGWTAYGTRGGWATG	YTTNGGYTTRTTKCCHGGGTCTC
PP61	DGARTAYGARAAAACATTTWTRCC	RATATAWACAWAVGGDATWC
PP63	RGAATATGARAARACRYTBATGCC	RTGDCCKATTTGYTGWGGATTY
PP64	TCCACAYGATYTAGARTATGARAARAC	YAWAWACATATGGAATTCT
PP65	AGAATATGARAAAACWTTTYATCC	TTCTATCTGCYAATACWGCRTGHGCT
PP66	WCCWCATGATTTRGARTATGAAAAA	WGCHARNACTTTRTGTGCWAT
PP67	WCAYGATYTDGAATAYGAAAARAC	RCWARARCADYATGAGCWAT
PP68	YGTAGGAATKCTWTATGAGSAK	YGGWTTBGGTTTATTYCCYGGW
PP69	WCCWCATGATTTRGAATATGARAA	WGCTARWACHTTATGRGCAATB
PP70	WCAHGWWYTAGAATATGARAAAAC	GGTTTATTWCCVGRATCHCKTTYACC
PP71	TCCWCAHGAYTTAGARTATGARAARAC	WGGWGCAYTWCCWKGATCTCTY
PP72	AAAARCGMTATGTYGGWATGY	MCCWGGYWTGGGTTTATTRCC
PP73	RARCATTSTATWMAATGTGGR	HGCVATACYYTKTGGATTTTTATAAWAT
PP74	WAARAARMGATATGTWGGDMAT	KATCWGCYAAWACMKYATGWGC
PP75	RGAATATGARAARACWTWYTGGCC	RACWACRTGASCWATTCKMSTCCAATC
PP76	RAGRTATRTTGGWAAYAAATATGAR	YGATTKWGGTTTRTTWCCTGGR
PP77	DCCACAYSAYTTRGARTATGARAA	BATVCGRTYCGCCAAYAC
PP78	KTATGARAAGRYATWWTWWCCATTT	MAGCWARYAYWWYATGAGC
PP79	RAGATAYRYWGGTWWWAAATATGAA	KTWGGWKYATTWCCTGGATC
PP80	CTTTACCCGTGCTTATTTC	GTGGTTGATTTTCAGGTG
PP81	WCCWCATRAACTCGAATATGA	YGCTAARRCKKYATGACCTATYTGC
PP82	WCCWCATAAAYTRGARTATGAAAAR	SCCATWCKRTCHGCTAATAYTTTATGW
PP83	CAAAATGTWCCHAAACCRCARAGT	ACTTTATGRGCAATWGTKGATGGTT

PP62 was not specific to 'Megaviridae' and thud excluded (see Materials and Methods for detail).

Table S3. *In silico* detection of ‘Megaviridae’ *polB* by MEGAPRIMER.

Name	Viruses included in the design of primers																	Viruses not included in the design of primers			
	CeV	CroV	OLPV1	OLPV2	PgV	PoV	APMV	hirudovirus_Sangsue	megavirus_bus	megavirus_courdo7	moumouvirus_M10A	moumouvirus_monve	moumouvirus_ochan	pointerouge1	terra2	megavirus_chiliensis	megavirus_lba	AaV	BsV	KNV1	CTV1
PP1																					
PP2									x	x						x	x	x			
PP3		x																			
PP4		x																			
PP5					x	x															
PP6																					
PP7																					
PP8																					
PP9		x																			
PP10																					
PP11																					
PP12	x	x																			
PP13																					
PP14																					
PP15																					
PP16	x		x																		
PP17	x																				
PP18		x		x																	
PP19	x				x																
PP20	x			x																	
PP21																					
PP22					x																
PP23	x																				
PP24	x																				
PP25	x				**X**																
PP26					x																
PP27																					
PP28			**X**	x														x			
PP29		x																			
PP30																					
PP31																					
PP32																					
PP33																					
PP34	x				x	x															
PP35				x														x			
PP36				x																	
PP37																					

PP38		x																			
PP39				x																	
PP40																					
PP41																					
PP42					x																
PP43																					
PP44					x																
PP45																					
PP46																					
PP47		X				X	x	x	x	x				x	x	x	x		x	x	
PP48																					
PP49																				x	
PP50					x																
PP51																					
PP52				x																	
PP53																					
PP54	x				x	x															
PP55																					
PP56																					
PP57				X																	
PP58																					
PP59	x																				
PP60				x																	
PP61			X															x			
PP63	x																				
PP64																					
PP65																					
PP66																					
PP67						x												x			
PP68																					
PP69						x															
PP70	X	x																			
PP71		x																			
PP72	x																				
PP73																					
PP74							x	x	x	x				x	x	x	x		x		
PP75																					
PP76		x																			
PP77																					
PP78																					
PP79		x																			
PP80																					
PP81																					
PP82																					
PP83							X	X	X	X	X	X	X	X	X	X	X				

X: *In silico* perfect match between the primer target sequences.
x: *In silico* PCR detection with 10% of maximum mismatches allowed.
Green background represents genes which were not included in the MEGAPRIMER design.

Table S4. Number of genotypes of 'Megaviridae' from *Tara* Oceans metagenomic data.

Station	Depth	Size Fraction (µm)	Number of Genotypes
4	DCM	0.2-1.6	53
4	SUR	0.2-1.6	29
7	DCM	0.2-1.6	35
7	SUR	0.2-1.6	20
9	DCM	0.2-1.6	31
9	SUR	0.2-1.6	58
18	DCM	0-0.2	5
18	DCM	0.2-1.6	18
18	SUR	0.2-1.6	16
18	SUR	0-0.2	4
22	SUR	0-0.2	5
23	DCM	0.2-1.6	7
23	DCM	0-0.2	6
23	SUR	0.2-1.6	3
25	DCM	0-0.2	5
25	DCM	0.2-1.6	26
25	SUR	0.2-1.6	37
25	SUR	0-0.2	4
30	DCM	0.2-1.6	25
30	DCM	0-0.2	4
30	SUR	0.2-1.6	73
31	SUR	0-0.2	2
31	SUR	0.2-1.6	18
32	DCM	0-0.2	0
32	DCM	0.2-1.6	22
32	SUR	0-0.2	6
32	SUR	0.2-1.6	28
33	SUR	0.2-1.6	12
34	DCM	0-0.2	10
34	DCM	0.2-1.6	36
34	SUR	0.1-0.2	32
34	SUR	0-0.2	12
34	SUR	0.2-1.6	17
36	DCM	0-0.2	2
36	DCM	0.2-1.6	54
36	SUR	0-0.2	0
36	SUR	0.2-1.6	36
36	SUR	0.1-0.2	3
37	OMZ	0.2-1.6	30
37	OMZ	0.1-0.2	76
38	DCM	0-0.2	10
38	DCM	0.2-1.6	47
38	OMZ	0.1-0.2	8
38	OMZ	0.2-1.6	9
38	SUR	0.1-0.2	2

38	SUR	0-0.2	3
38	SUR	0.2-1.6	32
39	DCM	0-0.2	9
39	DCM	0.2-1.6	27
39	OMZ	0.2-1.6	16
39	OMZ	0.1-0.2	33
39	SUR	0-0.2	9
39	SUR	0.1-0.2	2
41	DCM	0-0.2	18
41	DCM	0.2-1.6	58
41	SUR	0.1-0.2	1
41	SUR	0.2-1.6	20
41	SUR	0-0.2	4
42	DCM	0.2-1.6	35
42	DCM	0-0.2	17
42	SUR	0.2-1.6	26
42	SUR	0-0.2	8
45	SUR	0.2-1.6	14
46	SUR	0-0.2	9
48	SUR	0.1-0.2	6
48	SUR	0.2-1.6	17
52	DCM	0.2-1.6	28
52	DCM	0-0.2	12
52	SUR	0.2-1.6	8
56	MES	0.2-3	6
56	SUR	0.2-3	12
57	SUR	0.2-3	15
58	DCM	0.2-3	44
62	SUR	0.2-3	12
64	DCM	0.1-0.2	12
64	DCM	0-0.2	7
64	DCM	0.2-3	52
64	MES	0.2-3	1
64	SUR	0-0.2	10
64	SUR	0.2-3	55
65	DCM	0.2-3	38
65	DCM	0.1-0.2	30
65	DCM	0-0.2	14
65	MES	0.2-3	4
65	SUR	0-0.2	13
65	SUR	0.1-0.2	29
65	SUR	0.2-3	35
66	DCM	0.2-3	34
66	DCM	0-0.2	8
66	SUR	0-0.2	9
66	SUR	0.2-3	58
67	SUR	0.45-0.8	93

67	SUR	0.2-0.45	289
67	SUR	0-0.2	9
67	SUR	0.2-3	67
68	DCM	0.2-0.45	36
68	DCM	0.45-0.8	30
68	DCM	0.2-3	27
68	DCM	0-0.2	9
68	MES	0.45-0.8	8
68	MES	0.2-3	6
68	SUR	0.45-0.8	36
68	SUR	0.2-3	30
68	SUR	0-0.2	7
68	SUR	0.2-0.45	51
70	MES	0.2-3	1
70	MES	0.45-0.8	7
70	MES	0-0.2	0
70	MES	0.2-0.45	8
70	SUR	0.2-0.45	44
70	SUR	0.2-3	15
70	SUR	0.45-0.8	30
70	SUR	0-0.2	6
72	DCM	0-0.2	3
72	DCM	0.2-3	41
72	MES	0.2-3	7
72	MES	0-0.2	1
72	SUR	0.2-3	41
72	SUR	0-0.2	2
76	DCM	0.2-0.45	45
76	DCM	0.2-3	20
76	DCM	0-0.2	1
76	DCM	0.45-0.8	17
76	MES	0.45-0.8	3
76	MES	0.2-3	1
76	SUR	0.2-3	11
76	SUR	0-0.2	2
76	SUR	0.2-0.45	67
76	SUR	0.45-0.8	19
78	DCM	0.2-3	19
78	DCM	0.2-0.45	55
78	DCM	0.45-0.8	21
78	MES	0.2-3	2
78	MES	0.45-0.8	1
78	SUR	0.2-0.45	62
78	SUR	0.2-3	30
78	SUR	0-0.2	1
78	SUR	0.45-0.8	18
82	DCM	0.2-3	37

82	DCM	0-0.2	1
82	SUR	0.2-3	26
84	SUR	0.2-3	24
85	DCM	0-0.2	2
85	DCM	0.2-3	30
85	MES	0.2-3	8
85	SUR	0.2-3	14
93	DCM	0.2-3	28
93	SUR	0.2-3	33
94	SUR	0.2-3	47
96	SUR	0.2-3	16
98	DCM	0.2-3	11
98	MES	0.2-3	5
98	SUR	0.2-3	14
99	SUR	0.2-3	21
100	DCM	0.2-3	18
100	MES	0.2-3	14
100	SUR	0.2-3	39
102	DCM	0.2-3	118
102	MES	0.2-3	6
102	SUR	0.2-3	65
109	DCM	0.2-3	37
109	DCM	0-0.2	14
109	MES	0.2-3	6
109	SUR	0.2-3	38
109	SUR	0-0.2	8
110	DCM	0.2-3	38
110	MES	0.2-3	13
110	SUR	0.2-3	22
111	DCM	0.2-3	25
111	MES	0.2-3	5
111	SUR	0.2-3	36
112	DCM	0.2-3	23
112	MES	0.2-3	5
112	SUR	0.2-3	22
122	DCM	0.2-0.45	56
122	DCM	0.2-3	41
122	DCM	0.45-0.8	16
122	DCM	0.1-0.2	11
122	MES	0.1-0.2	2
122	MES	0.2-0.45	4
122	MES	0.2-3	9
122	MES	0.45-0.8	2
122	SUR	0.2-3	27
122	SUR	0.45-0.8	17
122	SUR	0.2-0.45	28
122	SUR	0.1-0.2	12

123	DCM	0.45-0.8	6
123	DCM	0.1-0.2	16
123	DCM	0.2-0.45	25
123	DCM	0.2-3	37
123	SUR	0.45-0.8	32
123	SUR	0.2-3	43
123	SUR	0.2-0.45	41
124	DCM	0.45-0.8	15
124	DCM	0.2-3	30
124	DCM	0.1-0.2	18
124	DCM	0.2-0.45	33
124	SUR	0.45-0.8	11
124	SUR	0.2-0.45	26
124	SUR	0.1-0.2	13
124	SUR	0.2-3	83
125	DCM	0.1-0.2	16
125	DCM	0.45-0.8	23
125	DCM	0.2-3	38
125	DCM	0.2-0.45	62
125	SUR	0.2-3	28
125	SUR	0.1-0.2	20
125	SUR	0.2-0.45	38
125	SUR	0.45-0.8	18
128	DCM	0.2-3	16
128	SUR	0.2-3	16
132	DCM	0.2-3	40
132	MES	0.2-3	4
132	SUR	0.2-3	25
133	DCM	0.2-3	132
133	MES	0.2-3	10
133	SUR	0.2-3	114
137	DCM	0.2-3	24
137	MES	0.2-3	23
137	SUR	0.2-3	50
138	DCM	0.2-3	45
138	MES	0.2-3	19
138	SUR	0.2-3	20
140	SUR	0.2-3	36
141	SUR	0.2-3	4
142	DCM	0.2-3	18
142	MES	0.2-3	6
142	SUR	0.2-3	31
145	MES	0.2-3	7
145	SUR	0.2-3	64
146	MES	0.2-3	2
146	SUR	0.2-3	39
148	MES	0.2-3	15

148	SUR	0.2-3	29
149	MES	0.2-3	1
149	SUR	0.2-3	64
150	DCM	0.2-3	42
150	SUR	0.2-3	50
151	DCM	0.2-3	34
151	SUR	0.2-3	38
152	DCM	0.2-3	57
152	MES	0.2-3	4
152	SUR	0.2-3	56

Table S5. Number of sequences and OTUs for individual primer pairs.

Name	Raw	High quality	With primers	Merged	BLASTP validated	Pplacer validated	Singleton OTUs (97%)	Non-singleton OTUs (97%)
PP1	43,872	41,934	36,981	24,482	2,754	2,751	8	48
PP2	322,013	321,254	315,500	310,568	303,992	303,898	309	290
PP4	597,319	41,780	37,369	31,151	24,358	24,346	81	233
PP5	70,359	70,266	68,285	59,566	49,132	46,085	112	195
PP8	109,407	34,185	19,080	16,120	13,181	13,180	62	161
PP9	538,706	17,708	13,152	10,991	9,124	9,097	64	175
PP10	215,185	75,246	47,920	41,061	32,507	32,470	37	145
PP12	261,662	195,848	112,926	98,109	92,781	92,512	267	685
PP13	731,861	30,055	28,083	27,680	26,844	26,842	2	2
PP14	98,336	71,647	64,726	58,511	52,768	52,322	40	195
PP15	178,077	134,822	116,239	107,505	85,655	85,654	82	204
PP16 26 cycles	26,839	26,457	26,219	25,624	24,938	24,706	161	566
PP16	148,545	147,810	146,433	139,153	133,588	132,792	495	779
PP16 38 cycles	321,645	316,612	313,377	305,423	295,745	293,415	345	929
PP17	98,882	73,694	54,856	27,696	3,775	3,775	17	99
PP18	185,579	184,370	182,867	176,711	169,404	169,281	317	890
PP19	93,336	93,138	88,070	61,132	21,495	21,494	35	173
PP20	304,295	27,039	17,928	11,889	5,281	5,281	54	123
PP23	24,585	20,217	17,775	10,420	5,302	5,300	48	122
PP24	82,131	80,884	78,916	48,197	41,200	40,392	983	551
PP25	65,677	65,582	63,464	53,890	44,944	43,149	77	299
PP26	173,125	149,688	141,845	133,441	124,780	124,754	217	632
PP27	356,662	134,773	124,016	119,497	114,044	110,853	105	361
PP29	372,344	353,711	327,753	313,388	285,086	284,986	173	414
PP31	59,921	59,055	53,804	37,489	16,488	16,488	40	223
PP33	493,259	416,575	151,951	139,666	118,349	118,325	102	299
PP34	235,134	235,102	228,534	219,839	208,770	204,282	243	431
PP35	532,470	528,309	523,716	512,130	480,637	480,545	454	516
PP37	316,988	315,876	283,597	268,445	252,261	252,227	79	123
PP38	192,334	191,155	187,275	174,145	154,464	152,682	191	666
PP40	369,144	267,559	246,382	229,511	215,728	215,708	146	611

PP41	678,171	13,430	6,947	5,195	3,290	3,224	21	84
PP44	665,669	32,899	28,608	26,605	24,913	24,913	82	269
PP45 26 cycles	73,314	26,316	25,604	25,105	24,540	24,399	160	444
PP45	297,664	272,454	268,975	262,928	256,846	256,248	407	615
PP45 38 cycles	409,219	334,953	328,313	316,745	309,040	307,426	575	781
PP46	786,086	289,071	280,202	273,924	268,041	268,007	193	249
PP48	379,717	379,532	368,541	358,829	345,130	344,656	127	142
PP50	124,350	123,570	116,265	109,729	101,145	95,352	117	327
PP51	586,019	148,681	139,755	135,437	130,944	130,770	81	216
PP52	243,093	212,721	175,017	141,589	130,459	130,459	64	83
PP53	63,638	59,240	53,975	43,463	29,427	29,143	80	334
PP54	69,328	67,433	54,882	50,307	46,622	46,601	185	438
PP55	449,690	448,173	415,028	397,463	380,952	380,874	429	585
PP56	371,093	370,911	368,552	361,286	352,724	352,713	115	99
PP59	89,181	88,411	81,889	73,473	64,786	64,781	79	198
PP60	288,054	246,475	216,665	158,322	113,225	113,158	156	450
PP61	207,927	198,608	194,626	170,486	100,622	100,621	103	155
PP63	221,599	207,982	159,181	108,750	61,244	61,242	42	147
PP64	289	264	0	0	0	0	0	0
PP65	236,953	236,929	226,396	173,419	107,574	107,429	87	167
PP66	245,644	245,457	243,349	239,937	234,839	234,836	193	513
PP68	172,191	171,057	161,076	141,575	123,454	118,948	40	62
PP69	563,995	560,072	542,589	531,260	517,549	517,425	536	840
PP70	434,505	434,364	418,359	407,478	396,705	396,088	378	1,000
PP71	379,063	378,541	367,173	354,991	328,867	328,545	467	931
PP72	180,810	124,659	99,784	67,781	52,501	52,347	62	155
PP73	358,612	345,758	339,850	331,234	322,674	322,661	442	181
PP75	249,269	249,162	221,694	149,000	83,723	83,723	31	103
PP77	82	79	0	0	0	0	0	0
PP81	65,776	65,120	27,228	9,120	30	30	0	1
PP82	166,802	163,086	162,136	158,609	154,284	154,275	149	261
